# Population genetic structure and direct observations reveal sex-reversed patterns of dispersal in a cooperative bird

**DOI:** 10.1111/mec.12978

**Published:** 2014-11-15

**Authors:** Xavier A Harrison, Jennifer E York, Andrew J Young

**Affiliations:** * Zoological Society of LondonRegent's Park, London, NW1 4RY, UK; † Centre for Ecology & Conservation, University of ExeterCornwall Campus, Penryn, TR10 9FE, UK

**Keywords:** corrected assignment index, FST, isolation by distance, sex-biased dispersal, spatial autocorrelation analysis

## Abstract

Sex-biased dispersal is pervasive and has diverse evolutionary implications, but the fundamental drivers of dispersal sex biases remain unresolved. This is due in part to limited diversity within taxonomic groups in the direction of dispersal sex biases, which leaves hypothesis testing critically dependent upon identifying rare reversals of taxonomic norms. Here, we use a combination of observational and genetic data to demonstrate a rare reversal of the avian sex bias in dispersal in the cooperatively breeding white-browed sparrow weaver (*Plocepasser mahali*). Direct observations revealed that (i) natal philopatry was rare, with both sexes typically dispersing locally to breed, and (ii), unusually for birds, males bred at significantly greater distances from their natal group than females. Population genetic analyses confirmed these patterns, as (i) corrected Assignment index (AIc), *F*_ST_ tests and isolation-by-distance metrics were all indicative of longer dispersal distances among males than females, and (ii) spatial autocorrelation analysis indicated stronger within-group genetic structure among females than males. Examining the spatial scale of extra-group mating highlighted that the resulting ‘sperm dispersal’ could have acted in concert with individual dispersal to generate these genetic patterns, but gamete dispersal alone cannot account entirely for the sex differences in genetic structure observed. That leading hypotheses for the evolution of dispersal sex biases cannot readily account for these sex-reversed patterns of dispersal in white-browed sparrow weavers highlights the continued need for attention to alternative explanations for this enigmatic phenomenon. We highlight the potential importance of sex differences in the distances over which dispersal opportunities can be detected.

## Introduction

Dispersal is a fundamental process in ecology that has a profound influence at multiple levels of organization, from the reproductive success of individuals to the genetic structure and viability of populations. A key, unresolved question in evolutionary ecology is why dispersal is so commonly sex-biased (where one sex disperses further, or at a higher rate, than the other) and, furthermore, why some species show male-biased dispersal (e.g. the majority of mammals; Greenwood 1980), while in others dispersal is female-biased (e.g. the vast majority of passerine birds; Greenwood 1980; Clarke *et al.* 1997). A multitude of hypotheses have been proposed to explain the evolution of sex-biased dispersal and the direction of any sex bias, including roles for inbreeding avoidance (Pusey 1987; Clutton-Brock 1989; Perrin & Mazalov 2000), local competition for mates or resources (Greenwood 1980; Perrin & Mazalov 2000), local resource enhancement (Perrin & Mazalov 2000), parent–offspring conflict (Waser & Jones 1983; Liberg & von Schantz 1985) and sex differences in the relative importance of breeding opportunities within and outside the natal group (the ‘breeding diversity’ hypothesis; Yáber & Rabenold 2002). Despite intense research effort over the past four decades, the primary evolutionary drivers of sex-biased dispersal remain hotly debated (Lawson Handley & Perrin 2007; Clutton-Brock & Lukas 2012; Mabry *et al.* 2013; Shaw & Kokko 2014). Our ability to evaluate competing hypotheses for the evolution of sex-biased dispersal hinges upon our ability to (i) accurately quantify its magnitude and direction in natural populations (Koenig *et al.* 1996; Clarke *et al.* 1997; Prugnolle & de Meeus 2002) and (ii) identify model species that show reversals of the taxonomically conserved norms for the direction of any sex bias (e.g. rare examples of male-biased dispersal in passerine birds; Williams & Rabenold 2005; Berg *et al.* 2009). Such species with sex-reversed patterns of dispersal offer the greatest potential for testing evolutionary hypotheses for dispersal sex biases (Greenwood 1980; Langen 1996; Berg *et al.* 2009; Lawson Handley & Perrin 2007; Clutton-Brock & Lukas 2012; Dobson 2013), both through targeted work on these model systems (e.g. Langen 1996; Williams & Rabenold 2005; Berg *et al.* 2009) and their inclusion in comparative analyses whose power is otherwise constrained by the rarity of such reversals (e.g. Mabry *et al.* 2013).

Obtaining accurate, unbiased estimates of dispersal can be problematic. Direct observations of dispersal events in the wild can be time-consuming and logistically challenging to obtain, especially for cryptic species, and can underestimate true dispersal because of a bias towards detecting short-distance dispersal events (Koenig *et al.* 1996). Dispersal values can be corrected for such bias (e.g. Sharp *et al.* 2008), but the accuracy of the corrected estimates declines as the difference between the observed and true maximal dispersal distance increases (Koenig *et al.* 1996), leaving corrected estimates potentially unreliable if the observational data are sparse. Collecting dispersal observations over comparatively short time periods can also leave the resulting estimates of dispersal patterns vulnerable to transient sex biases that may not be representative of longer-term norms (Pérez-González & Carranza 2009; Eikenaar *et al.* 2010). Indirect, genetic methods offer an alternative means of quantifying dispersal that avoids the spatial biases associated with observational data and may be more indicative, in long-lived species, of the long-term average pattern of sex-biased dispersal (e.g. Goudet *et al.* 2002; Peakall *et al.* 2003; Banks & Peakall 2012). However, previous studies have demonstrated that estimates based on genetic data alone can differ from estimates derived from observational data (Winters & Waser 2003; Lukas *et al.* 2005; Harris *et al.* 2009; Rollins *et al.* 2012). Such differences may arise in part because indirect genetic methods examine the population genetic patterns arising from both the permanent dispersal of individuals and the spread of gametes (e.g. in species that temporarily move to mate; Waser & Elliott 1991; Double *et al.* 2005; Griesser *et al.* 2013), and in some cases, the genetic signature of gamete dispersal can shroud or exaggerate that of individual dispersal (Winters & Waser 2003). The most robust estimates of sex differences in dispersal can thus be derived using a combination of direct observational data and indirect population genetic methods (e.g. Ribeiro *et al.* 2012; Rollins *et al.* 2012), integrating where possible information on spatial patterns of extra-pair mating to account for gamete dispersal (e.g. Double *et al.* 2005; see also Smouse & Peakall 1999; Vekemans & Hardy 2004 for gamete dispersal in plants).

In cooperatively breeding species, characterizing the nature of any sex difference in dispersal is especially important for understanding localized patterns of intrasexual kin structure and, by extension, sex-specific patterns of cooperation and conflict (Johnstone & Cant 2008; Gardner 2010; Young & Bennett 2013). While some cooperatively breeding birds show no clear sex bias in dispersal (e.g. Eikenaar *et al.* 2010; Blackmore *et al.* 2011; Nelson-Flower *et al.* 2012), the majority show the typical avian sex bias; females are more likely to disperse from their natal groups than males and/or do so over greater distances than males (Greenwood 1980; Stacey & Koenig 1990; Clarke *et al.* 1997), frequently yielding higher levels of both within-group and ‘neighbourhood’ kinship among males (Hatchwell 2010). However, a handful of cooperatively breeding bird species are unusual among passerines in that males appear to be the more dispersive sex (white-throated magpie jay, *Calocitta formosa*, Langen 1996; Berg *et al.* 2009; brown jay, *Cyanocorax morio*, Williams & Rabenold 2005; American crow, *Corvus brachyrhynchos hesperis*, Caffrey 1992; Australian magpie, *Gymnorhina tibicen*, Veltman & Carrick 1990; Hughes *et al.* 2003; see Eikenaar *et al.* 2010 for no clear long-term dispersal sex bias in the Seychelles warbler, *Acrocephalus seychellensis*, in which early evidence suggested male-biased dispersal; Richardson *et al.* 2002). While these few species, all members of the *Corvoidea* superfamily, have offered new insights into the potential drivers of dispersal sex biases in animal societies (Langen 1996; Yáber & Rabenold 2002; Williams & Rabenold 2005; Berg *et al.* 2009; see Discussion), attempts to identify generalities demand the identification and examination of additional reversals in cooperatively breeding birds from other taxonomic groups.

Here, we combine direct longitudinal observations of dispersal with a cross-sectional analysis of population genetic structure to demonstrate a rare reversal of the typical avian sex difference in dispersal, in the cooperatively breeding white-browed sparrow weaver (*Plocepasser mahali*). White-browed sparrow weavers live in social groups comprising a dominant breeding pair and up to 12 helpers of approximately equal sex ratio (Harrison *et al.* 2013a). Recent genetic analyses have revealed that although the dominant pair monopolizes within-group reproduction (Harrison *et al.* 2013a), dominant males lose 12–18% of paternity to extra-group males (Harrison *et al.* 2013a,b). Both sexes of white-browed sparrow weaver frequently delay dispersal from their natal group well to adulthood and help to rear subsequent clutches of offspring from the dominant pair, typically their parents (Harrison *et al.* 2013a). However, individuals of both sexes do emigrate to either join existing social groups or find new territories as breeding pairs and/or mixed-sex trios (Lewis 1982a; Harrison *et al.* 2013a). Previous work on a more northerly subspecies (*P. m. pectoralis*) suggests that both sexes typically disperse to breed and that the majority of dispersal distances are relatively short (<500 m; Lewis 1982a). However, the sex-specific patterns of dispersal in this species remain unclear, due in part to the sexes of the *pectoralis* subspecies (unlike those of our focal subspecies, *P. m. mahali*) being morphologically indistinguishable in the field (Collias & Collias 1978; Lewis 1982b).

First, we use 5 years of observational data to (i) confirm the rarity of natal philopatry (inheritance of a dominant breeding position within the natal group) and (ii) establish the direction and magnitude of any sex difference in natal dispersal distance (the distance from birth to first obtaining a dominant breeding position; see methods), utilizing simulations to correct for detectability bias. Second, we use indirect genetic methods to draw inferences about sex differences in dispersal, by contrasting the sex-specific patterns of spatial genetic structure, using both population-level (e.g. assignment index and *F*_ST_ tests) and individual-level (i.e. spatial autocorrelation) analyses, and assess the congruence of the dispersal insights from this approach with those derived from the observational data. Finally, we examine the distribution of distances over which ‘sperm dispersal’ occurs in this population via extra-group mating, so as to clarify whether its contribution to spatial genetic structure could have lead to an over- or under-estimation of any sex difference in individual dispersal on the basis of the spatial genetic data alone. We close by then considering the potential for leading hypotheses for the evolution of dispersal sex biases in social species to account for the patterns observed.

## Methods

### Study population

The study population comprised 39 cooperatively breeding groups of white-browed sparrow weavers that defend year-round territories in an area of approximately 1.5 km^2^ in Tswalu Kalahari Reserve, South Africa (see Harrison *et al.* 2013a; Cram *et al.* 2014). The study population forms a single contiguous block of adjoining territories that has been continuously monitored for all breeding seasons (October–May) since 2007, such that any permanent movement of individuals into or within the study site would be detected, including transitions between established groups and the finding of new territories. The study population is surrounded in large part by elevated dunes that do not support sparrow weaver territories, but there are unmonitored territories within the known dispersal distance of the birds, and so, the study population does receive a small number of unmarked immigrants each year. Adult males and females can be readily distinguished from about 6 months of age as males have dark-brown beaks, while females have paler beaks. The dominant bird of each sex was determined by weekly monitoring of dominance-related aggressive, displacement and reproductive behaviours (details in Harrison *et al.* 2013a & York *et al.* 2014). All birds were fitted with a single metal ring and three colour rings for individual identification, under SAFRING licence 1444. All protocols were approved by the University of Pretoria Ethics Committee and complied with regulations stipulated in the Guidelines for Use of Animals in Research.

### Natal dispersal distance estimates

Following classical definitions of natal dispersal (e.g. Greenwood & Harvey 1982; ‘dispersal from the site or group of birth to that of first reproduction or potential reproduction’), we calculated natal dispersal distances as the Euclidean distance between an individual's natal group and the social group where it first attained a dominant breeding position (as the dominant male and female in each group completely monopolize within-group reproduction; Harrison *et al.* 2013a,b). Natal dispersals in our data set could therefore have arisen through two routes: (i) individuals that dispersed from their natal group and attained dominance in the first group in to which they dispersed and (ii) individuals that first dispersed to a group as a (nonbreeding) subordinate and subsequently dispersed again to attain their first dominant breeding position elsewhere. Focusing in this way on displacements from natal to breeding sites is of most relevance to (i) key hypotheses for the evolution of sex-biased dispersal (such as inbreeding avoidance or reproductive competition) and (ii) attempts to understand its population genetic consequences, as dispersals to nonbreeding positions that yield no descendents may have little downstream impact on population genetic structure (Yáber & Rabenold 2002; Griesser *et al.* 2013). Our data set comprises measures of natal dispersal distance for 33 birds (18 females & 15 males) originating from 18 unique natal groups, all of which occurred during the 5-year period between the breeding seasons of 2007/2008 and 2011/2012 inclusive. We assessed the significance of the sex difference in mean natal dispersal distance using a randomization approach. For each iteration, the sexes were randomly permuted among the distance observations, and the mean dispersal distance for each sex was calculated and stored. We performed a total of 10 000 iterations to build a null distribution of dispersal distances, and calculated 95% confidence intervals for the randomized *P* value following Ruxton & Neuhäuser (2013), as implemented in r v3.1.0 (R Core Team 2014).

### Testing nonrandom dispersal

We performed a simulation procedure to test whether the observed patterns of male and female dispersal were nonrandom with respect to distance within the bounds of the study site. The randomization procedure was conducted as follows: (i) for each natal dispersal event (representing a dominance turnover event in the destination group), we recorded the sex and destination group of the observed disperser, to be kept constant for all simulations; (ii) for each iteration, we randomly selected a new source group by selecting a natal subordinate of the same sex from one of the groups in the study site (excluding the destination group to ensure no philopatry); (iii) we calculated the distance between the destination group and the randomly chosen source group; (iv) we repeated this procedure for a total of 10 000 iterations to build a null distribution of sex-specific random dispersal; and (v) we compared the observed dispersal distance for each sex to the simulated values to derive a 2-tailed *P* value for the test of nonrandom dispersal, with the significance level set to 0.05. Each iteration utilized the 33 observed dispersal events (18 females and 15 males), and the mean observed dispersal distance for each sex was calculated as the mean value for all observed dispersal distances across all seasons in our data set (2007/2008–2011/2012).

### Correcting dispersal patterns for detectability

Estimates of dispersal distance can be downwardly biased by imperfect detection of long-distance dispersal events, with the probability of detection depending strongly on the size and shape of the monitored area (Koenig *et al.* 1996). We therefore corrected our dispersal estimates using a simulation procedure based on Sharp *et al*. (2008). We simulated 10 000 dispersal events for all distances at 20-m intervals between 60 and 1440 m inclusive (covering the full range of observed values in our data set). For each iteration, a random social group in the study site was chosen as a starting location and a random dispersal direction was chosen from a uniform distribution on the interval 0–359.99 degrees, with increments of 0.01. As our study population comprises a contiguous block of monitored territories, we assumed all simulated dispersal within the bounds of our study site had perfect detectability, meaning the probability of detection of a given dispersal distance is calculated as the proportion of simulated events that land within the study site. The estimate of the true number of recruits from our study population dispersing a given distance is then calculated as the inverse of the detection probability at that distance multiplied by the number of recruits from our study population detected as having dispersed that distance, allowing one to calculate corrected dispersal frequency histograms for each distance class (Baker *et al.* 1995; Sharp *et al.* 2008). To allow the prediction from our simulation outputs of a detection probability for *any* given dispersal distance, we modelled simulated detection probability as a function of distance using a binomial general linear model (glm) with a 2-column vector of number of detections: number of failed detections (from which detection probability can be calculated as a binomial response) and distance as a predictor. We allowed for both a linear and nonlinear effect of distance, using AICc to rank models with (i) a linear distance term; (ii) 2nd order polynomial for distance and (iii) 3rd order polynomial term for distance. We then used the best model to predict the detection probability for all of the natal dispersal distances observed in our data set.

### Genetic tests of dispersal

All genetic tests listed below use genotypes from 10 polymorphic microsatellite loci described in Harrison *et al*. (2013a) as genetic data. For spatially explicit genetic methods (isolation by distance and spatial autocorrelation analysis), we used GPS coordinates of roost trees in the centre of the territories of social groups (details in Harrison *et al.* 2013a) as the spatial location of genotypes within that social group. All analyses use a sample of 185 individuals known to be alive in the core study population on 1 January 2011 (an arbitrarily chosen date) to represent a cross-sectional sample of individuals and their spatial locations at a specific time. For in-depth reviews on the use of indirect genetic methods to quantify spatial genetic structure and dispersal, see Goudet *et al*. (2002); Prugnolle & de Meeus (2002); and Banks & Peakall (2012).

#### *F*_ST_ tests

We used the analysis of molecular variance (amova) framework in genalex v6.5 (Peakall & Smouse 2006, 2012) to calculate Wright's F statistics (Wright 1931). In the case of cooperative breeders, the *F*_ST_ statistic represents the proportion of genetic variance that is partitioned among different social groups. Low *F*_ST_ values imply that social groups are genetically homogenous, whereas high values suggest that social groups represent genetically distinct units of individuals, and the lower the rate of migration among social groups, the higher the *F*_ST_ value. Sex-biased dispersal can be assessed by calculating *F*_ST_ separately for males and females, as under conditions of sex-biased dispersal, the more philopatric sex is expected to show higher *F*_ST_ values (Goudet *et al.* 2002). Samples sizes for this analysis, drawn from the pool of 185 individuals, alive in the population as of 1 January 2011 were the following: males – 84 individuals from 30 groups containing at least two males and females: 74 individuals from 22 groups containing at least two females. Significance of differences in *F*_ST_ between males and females was tested by permutation analysis following the procedures implemented in fstat by Goudet *et al*. (2002).

#### Assignment tests

Assignment indices quantify the probability that a genotype originated in the population from which it was collected and therefore can function to distinguish immigrants from residents (Favre *et al.* 1997; Prugnolle & de Meeus 2002). Population effects are removed by subtracting the population mean assignment index from each individual assignment index, yielding a corrected assignment index for each genotype (AIc, Goudet *et al.* 2002). Strongly negative AIc values indicate the rarity of a given genotype and thus may reflect recent immigrant ancestry (Favre *et al.* 1997; Prugnolle & de Meeus 2002). Therefore, one expects that the more dispersive sex would, on average, possess lower AIc values than the philopatric sex. AIc calculations were carried out in fstat using the sample size detailed above for the *F*_ST_ calculations.

#### Spatial autocorrelation analysis

To examine sex differences in fine-scale spatial genetic structure in our study population, we performed a spatial autocorrelation analysis (SAA) as implemented in genalex v6.5 (Peakall & Smouse 2006, 2012). SAA is a multivariate method, utilizing data from all typed loci simultaneously to strengthen the signal of spatial structure by averaging over stochastic locus-to-locus variation (Smouse & Peakall 1999). The method requires two input matrices – a pairwise geographic distance and pairwise squared genetic distance matrix, both of which can be calculated from raw genotypic and spatial data entered into genalex using methods described in Smouse & Peakall (1999). Using these genetic and geographic distance matrices in conjunction with a user-specified distance class, SAA calculates an autocorrelation coefficient *r* among genotypes within each distance class, bounded by −1 and 1. When genotypic data are used as one of the input matrices, *r* is closely correlated with genetic relatedness (see Double *et al.* 2005; Blackmore *et al.* 2011). genalex uses bootstrapping to calculate 95% confidence intervals around the mean value of *r*, and permutation analysis (random sampling of individuals among groups) to calculate 95% confidence intervals around the null hypothesis of no genetic structure (Peakall *et al.* 2003). Significant genetic structure is indicated either when (i) mean *r* values fall outside the confidence intervals for the null model of no genetic structure or (ii) the 95% bootstrapped CIs around *r* do not cross zero. SAA methods can detect the occurrence of sex-biased dispersal because variation between males and females in patterns of dispersal (e.g. mean dispersal distance) is expected to produce different patterns of fine-scale spatial autocorrelation (Banks & Peakall 2012). For example, the least dispersive sex is expected to show significant, positive genetic structure at short-distance classes (e.g. because related individuals remain close to their natal groups), whilst the more dispersive sex often lacks significant structure at any distance class (for examples see Peakall *et al.* 2003; Double *et al.* 2005; Banks & Peakall 2012).

We conducted SAA in two discrete ways. First, to test the prediction that *within-group* genetic structure will be stronger in the sex demonstrating more restricted dispersal (e.g. higher intrasexual relatedness due to lower frequency of unrelated immigrants introducing dissimilar genotypes into groups), we quantified spatial genetic structure using all 185 individuals alive in 39 social groups as of 1 January 2011, a sample comprising both dominant and subordinate birds. Second, to test the prediction that the sex that demonstrates shorter natal dispersal distance should show stronger genetic structure at local distances (i.e. breeders in the same vicinity should be more similar to one another in the sex showing restricted dispersal), we quantified spatial genetic structure of only breeding individuals using the dominant birds alive as of 1 January 2011 (*n* = 39 dominant males and females). This approach more closely reflects the analysis of observational natal dispersal data, where we consider only individuals that have moved to take up breeding positions.

We used 250-m distance classes to represent a distance of 1–2 territories from a focal territory (mean distance between territory centres: 117 m, Harrison *et al.* 2013a). For the first analysis (using all 185 individuals), we set the first distance class to 0 to represent only within-group comparisons. For the second (dominants only) analysis, within-group same-sex comparisons were not possible and so the first distance class was set to 250 m. We specified the ‘multiple populations’ option where each sex was listed as a separate population to test for differences in genetic structure between males and females using the ‘T2’ statistic at each distance class (details Banks & Peakall 2012) as calculated by genalex.

To test the sensitivity of our choice of distance class for the second analysis, we performed a sensitivity analysis as detailed in Double *et al*. (2005). The SAA is repeated multiple times, but for each iteration, the size of the distance class is increased. The greatest distance class where the 95% bootstrapped confidence intervals around *r* does not overlap zero is considered the extent of detectable genetic structure (Double *et al.* 2005). Where the strength of genetic structure differs according to sex, it is expected that genetic structure will remain detectable over larger distance classes for the more philopatric sex (Double *et al.* 2005). We used only the dominant individuals for this analysis to prevent any sex differences in within-group genetic structure from influencing our results, and calculated structure separately for each sex. We used distance classes increasing in size by 100 m at each step from 0–250 m up to 0–750 m inclusive.

#### Isolation by distance

We used the genalex software to test isolation by distance (IBD) separately in both dominant males and females, using the ‘Mantel test’ option. We used the same pairwise genetic and pairwise geographic distance matrices as for the SAA above. Significance was assessed using 999 random permutations of the data, as performed by genalex. Under conditions of restricted dispersal, one would predict significant isolation by distance, whereby local genotypes are more similar to one another than more distant genotypes. With respect to sex-biased dispersal, one would expect the sex demonstrating more restricted dispersal to demonstrate IBD, whilst the more dispersive sex would exhibit either no IBD or weaker IBD.

#### Spatial patterns of extra-group paternity

We used data from 19 extra-group mating events for which the extra-group sires had previously been identified (see Harrison *et al.* 2013b), to assess the potential for the spatial patterns of extra-group mating to have influenced the sex-specific patterns of population genetic structure described by the analyses above. First, we performed a simulation procedure to test for nonrandom patterns of extra-group mating in space (i.e. whereby sparrow weavers show a tendency, for example, to conduct extra-group matings significantly closer to their home territories than would be expected by chance). For each iteration, we randomly chose a dominant male from one of the social groups present in the population at the time of each extra-group paternity for each of the 19 clutches and computed the distance between the extra-group-mating female and the randomly chosen dominant male. We then calculated and stored the mean and median of these 19 distances and performed 10 000 iterations in total. We used these stored values as a null distribution representing random extra-group mating to which we compared the true mean and median of the EGP data set. Second, we contrasted the distances over which extra-group matings occurred (which entail the dispersal of the gametes of males), with the distances over which males themselves engaged in natal dispersal.

## Results

### Observational evidence of sex-biased dispersal

**Fig 1:**
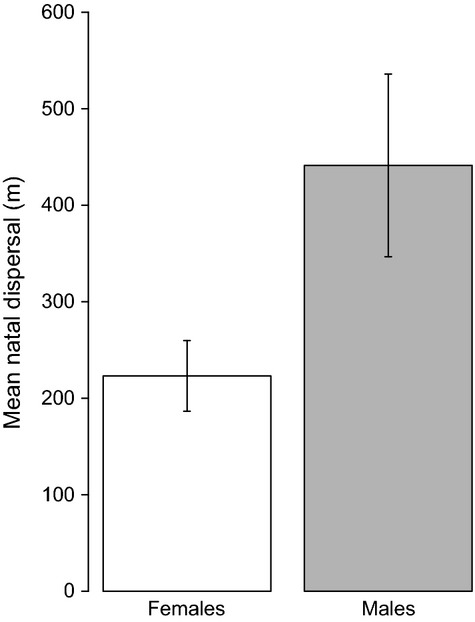
Mean uncorrected natal dispersal distance for males and females (the dispersal distance from their natal group to where they first attained a dominant breeding position). Bars present means ± SE estimated by bootstrapping.

Dominant breeding positions were rarely inherited by birds within their natal groups (8 of 54 dominance turnover events, 14.8%), and there was no clear sex difference in the incidence of doing so (3 of 25 (12.0%) female dominance turnovers; 5 of 29 males (17.2%); binomial test: 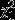 = 0.03, *P* = 0.88). Of the 46 dominance turnovers that did not involve inheritance within the natal group, 33 of the new dominants were known natal dispersers (i.e. they were known to be securing their first dominant position), two were known breeding dispersal events (i.e. the bird was previously dominant in another group), and for the remaining 11, the birds originated outside the study population and so could have been undertaking either natal or breeding dispersal. For the 33 known natal dispersal events, the natal dispersal distances (from the birds’ natal site to their first attainment of dominance) of males were significantly larger than those of females (mean ± SE males: 440.13 m ± 94.7; females: 223.28 ± 36.57; randomized *P* = 0.04, 95% CI = 0.037–0.046, Fig.1). As the mean distance between the centres of neighbouring territories in our population was 117 m (Harrison *et al.* 2013a), these translate into mean (± SE) detected natal dispersal distances of 3.78 (± 0.85) territories for males and 1.92 (0.34) territories for females. Simulations confirmed local dispersal by both sexes: the observed natal dispersal distances were significantly shorter than would be expected by chance if individuals were dispersing randomly with respect to distance within our study site (males: *P* = 0.003; females: *P* < 0.001). It seems unlikely that females frequently engage in a second long-distance dispersal strategy that has gone undetected due to the scale of our study site, as dominance positions within our study population were rarely secured by females originating outside it (just 4 of 25 dominance turnovers; 16.0%); the same was true for males (7 of 29; 24.1%).

**Fig 2:**
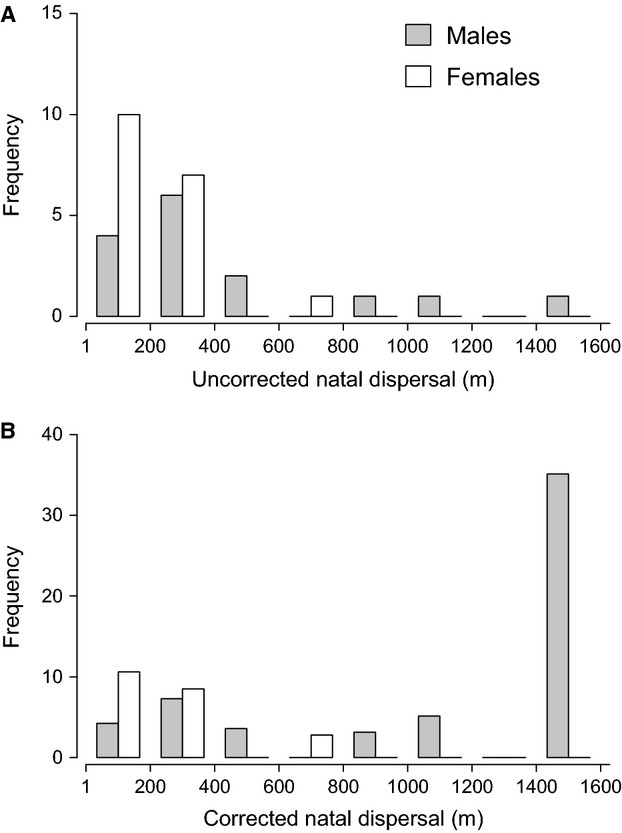
Natal dispersal distance histograms for males and females based on (A) observed data and (B) observed data corrected for sampling bias

As expected, the simulated dispersal detection probability declined significantly with distance (best supported model: 3rd order polynomial for distance, ΔAICc = 3910.8, Akaike weight = 1, Table S1 & Fig. S1, Supplementary information). Females typically undertook shorter natal dispersals than males (Fig.2A), which have a higher probability of detection (Fig. S1, Supplementary information). As a consequence, the increase in mean natal dispersal distance arising from correction was small for females (corrected female mean = 269.7 m, Δ from uncorrected mean = +46.4 m) compared to that for males (corrected male mean = 1069.69 m, Δ = +629.6). While the large correction increase for males was due in large part to a single highly weighted long-distance male dispersal (1429.5 m; Fig.2B), removing this data point still yielded a marked sex difference in the corrected breeding dispersal distances [corrected female mean = 269.7 m (approximately 2 territories); corrected male mean = 530.6 m (approximately 4 territories)].

### Population genetic evidence of sex-biased dispersal

#### Assignment indices

Corrected Assignment Index (AIc) values were significantly higher for females than males (female mean AIc 0.55 ± 0.19; males -0.66 ± 0.11; *P* = 0.003), indicating a higher incidence of rare genotypes among males than females, which is suggestive of higher rates of immigration into the study population among males than females.

#### *F*_ST_

Mean *F*_ST_ for the 39 social groups calculated using all 185 individuals was high (*F*_ST_ = 0.16, Table1), indicating significant genetic differentiation among groups, which is to be expected in this species as offspring delay dispersal. When calculating *F*_ST_ separately for each sex, females showed a higher mean *F*_ST_ value than males (females: 0.22, males: 0.15, Table1), indicating a significantly greater degree of genetic differentiation among groups for females than for males (fstat test: *P* = 0.039), a pattern consistent with males being the more dispersive sex.

**Table 1:** *F*_ST_ values from the population genetic analysis of the 185 white-browed sparrow weavers alive in the study population on 1 January 2011, split separately for males and females, and also for all individuals combined

Analysis	*N* groups	*N* ind.	Median & range per group	% Variation between groups	*F*_ST_	d.f.	*P*
Males	30	84	2.5 (2–5)	14	0.153	29	0.001
Females	22	74	3 (2–5)	20	0.221	21	0.001
Males & Females	39	185	5 (2–9)	15	0.157	38	0.001

‘*N* groups’: number of groups for each analysis. For single sex analyses, this is the number of groups containing at least 2 same-sex individuals, that is groups containing only a breeding pair were removed. ‘*N* ind.’: total number of individuals for each analysis; ‘Median & Range per Group’: the median, minimum and maximum number of individuals per group per analysis. ‘% Variation Between Groups’ and ‘*F*_ST_’: estimates of the amount of genetic variation partitioned among groups, where higher values indicate greater differentiation and reduced gene flow among groups.

#### Spatial autocorrelation analysis

**Fig 3:**
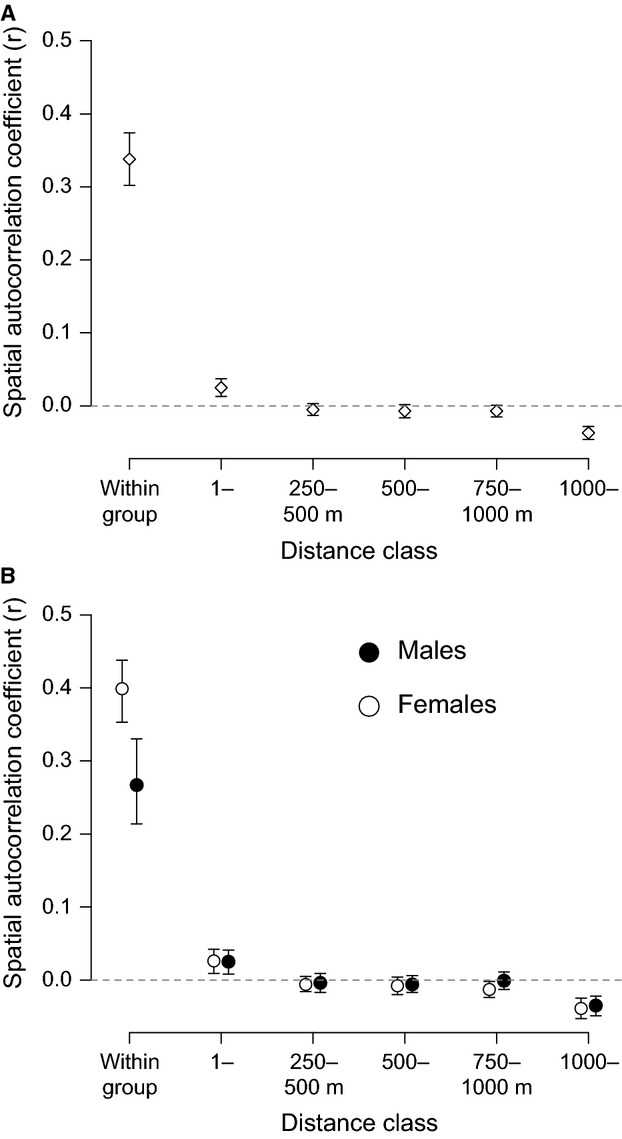
Population spatial genetic structure for 185 white-browed sparrow weavers in 39 social groups alive on 1 January 2011: (A) with both sexes combined and (B) calculated separately for each sex. Points represent the mean genetic spatial autocorrelation coefficient for that distance class. Error bars represent 95% confidence intervals around the mean estimate by bootstrapping. Error bars that do no overlap zero represent significant genetic structure.

Spatial autocorrelation analysis conducted on all 185 individuals from 39 groups (both sexes combined) revealed significant positive genetic structure both within groups and at the 250-m distance class (Fig.3A), indicating higher levels of allele sharing within these distances classes than would be expected under random mixing. When considering genetic structure separately for each sex, the within-group genetic correlation coefficient was significantly higher for females than for males (females = 0.35, males = 0.2, T2 = 13.64, *P* < 0.001). While both sexes still showed significant positive structure at 250 m, there were no sex significant differences in structure at this or any further distance class (all *P* > 0.183; Fig.3B).

**Fig 4:**
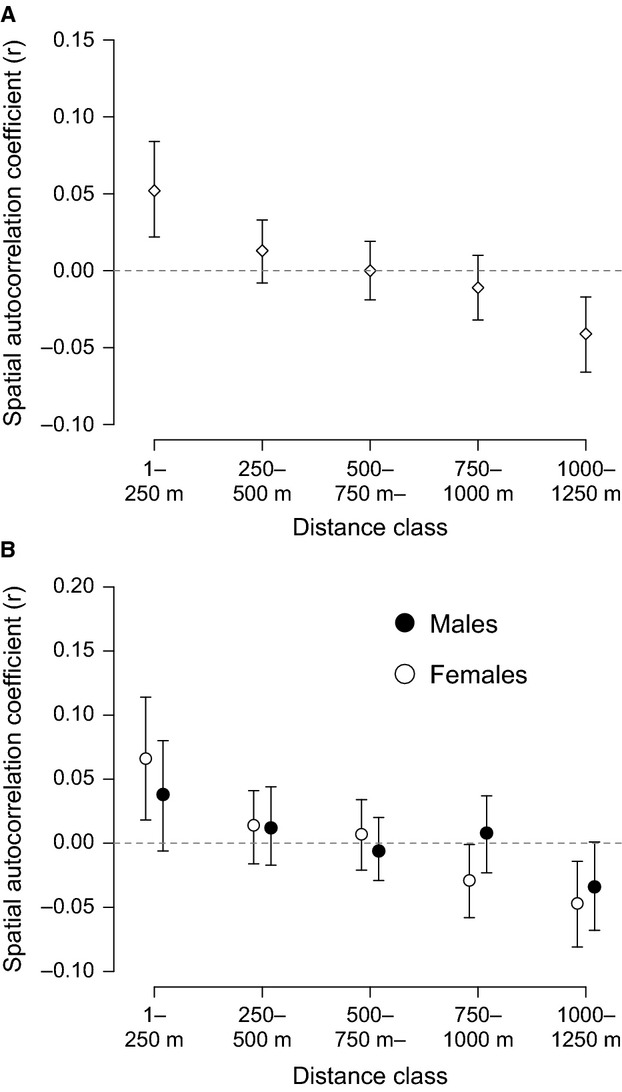
Population spatial genetic structure for 78 dominant (breeding) white-browed sparrow weavers from 39 social groups alive on 1 January 2011: (A) with both sexes combined and (B) calculated separately for each sex. Points represent the mean genetic spatial autocorrelation coefficient for that distance class. Error bars represent 95% confidence intervals around the mean estimate by bootstrapping. Error bars that do no overlap zero represent significant genetic structure.

**Fig 5:**
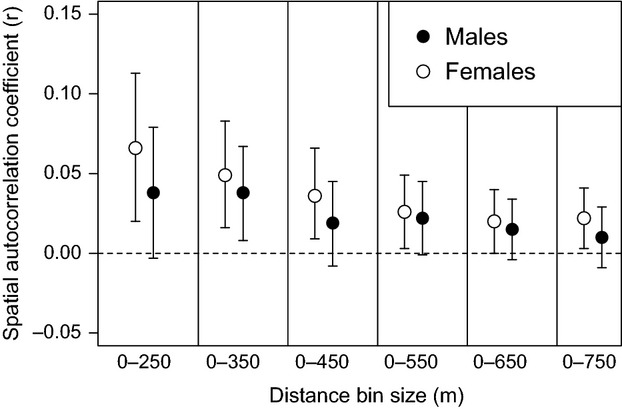
Sensitivity analysis for the genetic structure of dominant individuals showing the effect of different distance size classes used in the spatial autocorrelation analysis. Only the first distance class for each analysis is shown. 95% confidence intervals around mean r were estimated by bootstrapping. Dominant female positive genetic structure remained detectable using larger distance classes (up to 800 m) than for males (up to 600 m).

Repeating this analysis using only the dominant (breeding) male and female in each group yielded broadly similar results. Pooling both sexes, there was significant positive genetic structure in the 1- to 250-m interval, but no significant structure at longer distances (Fig.4A). There was no significant difference between males and females in the extent of genetic structure overall using this approach (Fig.4B; Ω  =  5.74, *P* = 0.49), although females were the only sex to show significant positive genetic structure in the 1- to 250-m interval. When performing the sensitivity analysis, significant positive local genetic structure for dominant females was detectable using distance bin sizes of up to 750 m, whereas for males, significant positive structure existed using only a 350-m bin size (Fig.5).

#### Isolation by distance

Both dominant males and dominant females showed significant genetic isolation by distance (males Rxy = 0.17, *P* = 0.001; females Rxy = 0.23, *P* = 0.001). As would be expected if males were the more dispersive sex, the slope of the relationship between distance and genetic similarity was steeper for females than for males (females: 0.0027; males: 0.0016), consistent with females showing a sharper increase in genetic dissimilarity with distance.

#### Spatial patterns of extra-group paternity

Randomization tests revealed that the distribution of distances over which extra-group mating occurred was consistent with random extra-group mating with respect to distance within the bounds of the study site (observed mean: 640.8 m, null distribution mean: 616.3 m, *P* = 0.72; observed median: 640.2 m, null distribution median: 592.3 m, *P* = 0.55). In addition, the distances over which extra-group mating occurred were significantly greater than those over which male natal dispersal occurred (*n* = 15 male natal dispersal events, median (interquartile range) = 277.5 (202.2–513.78) m; *n* = 19 extra-group mating events, median = 640.23 (347–513.78) m; *P* = <0.001, 95%CI <0.001–0.02).

## Discussion

Both the observational and genetic analyses conducted in this study are strongly suggestive of male-biased dispersal in white-browed sparrow weavers. This reflects a rare reversal of the typical avian pattern of female-biased dispersal (Greenwood 1980; Clarke *et al.* 1997; Mabry *et al.* 2013) and highlights an evolutionary origin for male-biased dispersal (in the superfamily *Passeroidea*) taxonomically distinct from the handful of known examples in cooperatively breeding birds (see Introduction). Observational data revealed that, while both sexes typically disperse to breed, females dispersed to take breeding positions at shorter distances from their natal groups than males, a contrast that became more striking on correction for detectability bias (following Koenig *et al.* 1996). Genetic data confirmed these patterns, with females showing both significantly higher mean *F*_ST_ values and corrected assignment indices (AIc) than males, both of which are indicative of male-biased dispersal. Spatial autocorrelation analysis confirmed the sex difference in genetic structure *within* groups, and, while there were no significant sex differences in the extent of structure outside groups, there was significant positive structure over greater distances among females than males and corresponding evidence suggestive of stronger genetic isolation by distance among females than males. That the average distances over which extra-group mating occurred exceeded the average male natal dispersal distance highlights the possibility that ‘sperm dispersal’ via extra-group mating may have acted in concert with individual dispersal to generate these sex-specific genetic patterns. However, such gamete dispersal alone cannot account entirely for the sex differences in genetic structure observed (see below). Our evidence of sex-reversed patterns of dispersal in this cooperative bird highlights a new model for evaluating (i) competing hypotheses for the evolution of dispersal sex biases and (ii) the evolutionary implications of dispersal sex biases in cooperatively breeding species. Below, we consider the evidence for local dispersal in both sexes and male-biased dispersal overall, before evaluating the extent to which leading hypotheses for the evolution of sex-biased dispersal can account for male-biased dispersal in white-browed sparrow weavers.

### Local dispersal and spatial genetic structure in both sexes

Both spatial autocorrelation analysis and *F*_ST_ values revealed strong signals of positive *within-group* genetic structure, which are indicative of high levels of relatedness within social groups, as would be expected of a species in which offspring of both sexes delay dispersal from their natal group (Harrison *et al.* 2013a; e.g. Beck *et al.* 2008). While high within-group relatedness could also be indicative of natal philopatry, in which offspring of one or both sexes frequently inherit the breeding position in their natal group, our observational data set revealed that such inheritance was comparatively rare for both sexes (see also Lewis 1982a), with no discernible sex bias in its likelihood. This is relatively unusual in social vertebrates, in which one sex may frequently inherit the breeding position on their natal territory, resulting in potentially long-term intrasexual dynasties distributed in space (among females: Clutton-Brock *et al.* 2002; Berg *et al.* 2009; Holekamp *et al.* 2012; among males: Cockburn *et al.* 2008; Walters *et al.* 2004). Indeed, the rarity of territorial inheritance by even the less dispersive sex in white-browed sparrow weaver societies might be expected to have diminished the long-term signal of sex differences in dispersal in this species’ population genetic structure (see below).

Multiple lines of evidence suggest that both sexes typically attain breeding positions close to their natal territories, resulting in kin neighbourhoods: (i) observational data revealed that the vast majority of detected natal dispersals occurred within 400 m (approximately 3 territories widths) of the natal group; (ii) simulations confirmed that natal dispersal movements for both sexes were significantly shorter than would be expected by chance if individuals dispersed randomly with respect to distance within the bounds of our study site; (iii) spatial autocorrelation analysis revealed positive, significant genetic autocorrelation coefficients for both males and females at the 250-m distance class; and iv) both sexes demonstrated significant isolation by distance, consistent with local dispersal. Similar patterns of local dispersal have been observed in other cooperatively breeding birds (e.g. Woxvold *et al.* 2006; Nelson-Flower *et al.* 2012; see also Lewis 1982a) and could be indicative of an adaptive response to a low turnover of breeding positions, whereby individuals delay dispersal and monitor for local vacancies from the safety of the natal territory, rather than risk longer-term prospecting over wider spatial scales for vacancies that may rarely become available (e.g. Lewis 1982a; Walters *et al.* 1992). Extra-territorial prospecting can be costly, often requiring otherwise social animals to traverse unfamiliar areas alone, exposing them to aggressive interactions, loss of body condition and the chronic elevation of stress hormones (Young *et al.* 2005; Ridley *et al.* 2008, Young & Monfort 2009), while also trading off against cooperative contributions that they might otherwise have made within their natal group (Young *et al.* 2005). Establishing or winning breeding positions close to the natal territory could also be facilitated if relatives within the natal group were more tolerant of such activities than nonrelatives elsewhere (e.g. if individuals attempted to annex a portion of the natal territory as their own independent breeding territory; Woolfenden & Fitzpatrick 1978; Kokko & Ekman 2002; Ekman *et al.* 2004; Hatchwell 2010) or if familiarity with individuals in the destination group facilitated immigration (Yáber & Rabenold 2002; Williams & Rabenold 2005). Local dispersal by both sexes may also entail fitness costs, however, arising from kin competition (Lehmann & Rousset 2010) and/or exposure to a risk of inbreeding (Koenig & Haydock 2004; Hatchwell 2010). Indeed, that there is overlap between the distributions of distances over which extra-group mating and dispersal occur may explain why extra-group matings in this population entail an elevated risk of inbreeding (Harrison *et al.* 2013b).

### Sex-biased dispersal and genetic structure

Together, our observational and genetic evidence indicates a reversal of the typical avian sex bias in dispersal (Greenwood 1980; Mabry *et al.* 2013). Our observational data reveal that, while both sexes typically disperse from their natal group to breed, males disperse significantly further to breed than females. The sex difference in dispersal distance became more pronounced following correction for detectability bias (following Koenig *et al.* 1996) as a higher proportion of male dispersals occurred over longer distances, with lower likelihoods of detection. The sex difference in our observational data arose principally from females, showing a modal natal dispersal distance of <200 m (frequently budding to establish a new territory on the edge of their natal territory or seizing dominance in a neighbouring group), while males showed a modal natal dispersal distance of 2–400 m (tending therefore to move just beyond their natal group's neighbours). Following the logic of Woolfenden & Fitzpatrick (1978), if larger sparrow weaver groups are better able to annex neighbouring habitat into which their resident females might disperse, subordinate females might stand to gain differential direct benefits from investing in group augmentation. As males are typically the more helpful sex in cooperatively breeding birds (Cockburn 1998; Clutton-Brock *et al.* 2002), the sex-reversed patterns of dispersal in this species might therefore be predicted to have yielded sex-reversed patterns of cooperation.

Several lines of evidence from the population genetic analysis support the observational evidence of male-biased dispersal in this species. *F*_ST_ analyses suggested a greater degree of between-group differentiation among females than males, whilst spatial autocorrelation analysis indicated a higher within-group similarity among females than males, both of which are consistent with the devaluation of spatial genetic structure among males caused by long-distance male dispersal (Goudet *et al.* 2002). In addition, Assignment Index tests revealed males to have significantly lower AIc scores than females, suggestive of novel/rare genotypes being more frequently introduced into the population through the long-distance dispersal of males (Goudet *et al.* 2002; Prugnolle & de Meeus 2002; see also Hansson *et al.* 2003). While spatial autocorrelation analysis *between social groups* did not reveal significant sex differences in spatial genetic structure, the gradient of genetic isolation by distance appeared to be steeper among females than males, and conducting SAA on only dominant (breeding) individuals revealed that only dominant females showed significant structure (at the 250-m distance class; males showed no significant structure at any distance class). Similarly, conducting a sensitivity analysis, following Double *et al*. (2005), revealed that genetic structure remained detectable over longer distances among dominant females than dominant males. While strong sex differences in dispersal would be expected to yield significant sex differences in spatial genetic structure among groups (as has been reported in superb fairy wrens, *Malurus cyaneus*, for example; Double *et al.* 2005), our findings echo simulation studies in suggesting that spatial autocorrelation analyses may struggle to detect more subtle sex differences in the incidence of dispersal or the distances over which it occurs (Banks & Peakall 2012). One factor that may temper the emergence of spatial genetic structure in sparrow weaver societies is the comparative rarity with which either sex inherits dominance within their natal group, such that (unlike in superb fairy wrens, for example, Cockburn *et al.* 2008) intrasexual dynasties of the less dispersive sex do not remain static in space over multiple generations.

The observed patterns of spatial genetic structure could also be driven partly by the occurrence of extra-group paternity. Dominant females monopolize 100% of reproduction and so are always parents of within-group offspring (Harrison *et al.* 2013a), whereas in approximately 15% of cases, dominant males lose paternity to extra-group males (Harrison *et al.* 2013b). The consequences of EGP are that (i) dominant males are not always related to within-group offspring and (ii) sets of maternal half-siblings are present in some groups (e.g. one offspring sired by the dominant within-group male and one offspring sired by an extra-group male). Male–male relatedness *within groups* will therefore be reduced, consistent with the SAA results, whilst genetic differentiation *among groups* will be reduced as a consequence of male gamete dispersal through promiscuity, consistent with results from the *F*_ST_ tests. However, evidence from the corrected Assignment Index tests, which revealed males to have significantly lower AIc scores than females, are unlikely to have been affected to the same degree by EG mating. The negative AIc values observed for males are suggestive of novel/rare genotypes being more frequently introduced into the population by males due to long-distance dispersal of unrelated immigrants (Goudet *et al.* 2002; Prugnolle & de Meeus 2002; see Dallimer *et al.* 2002 for AIc-based evidence suggestive of male-biased dispersal in another passerine). Long-distance EG mating could also be expected to introduce novel genotypes into the population, but would do so equally for the male and female offspring arising from EG matings and thus would be expected to reduce the assignment probabilities equally for both sexes. The negative AIc values we observe for males therefore most likely reflect the long-distance immigration of males, and not simply their gametes, a possibility further supported by the observational data set that suggested males are the sole sex that undertakes long-distance dispersals.

### The evolution of male-biased dispersal in white-browed sparrow weavers

Rare examples of male-biased dispersal in passerine birds provide valuable opportunities to evaluate the diverse competing hypotheses for the evolution of dispersal sex biases (Greenwood 1980; Langen 1996; Yáber & Rabenold 2002; Williams & Rabenold 2005; Berg *et al.* 2009; Mabry *et al.* 2013). Greenwood's (1980) seminal paper linked the directionality of the sex bias in dispersal in birds and mammals to mating systems. Where males show resource defence monogamy (as is frequently the case in birds), they were envisaged to benefit from defending resource territories in familiar habitat, close to their natal territory, while females may benefit from dispersal to choose the best males and/or territories, together resulting in female-biased dispersal (the typical avian pattern of dispersal). By contrast, where males show female defence polygyny (as is frequently the case in mammals), they were envisaged to benefit from dispersal to secure access to the largest number of females, resulting in male-biased dispersal (the typical mammalian pattern of dispersal). This perspective cannot readily account for the evolution of male-biased dispersal in white-browed sparrow weavers, however, as they do not exhibit female defence polygyny: like many cooperatively breeding passerines (Cornwallis *et al.* 2010), the dominant male and female form a largely monogamous pair (subject to 12–18% extra-group mating; Harrison *et al.* 2013a,b) and both sexes collectively defend a shared resource territory year-round (Wingfield & Lewis 1993). Indeed, a recent comparative study tested the extent to which mating system predicts the direction of dispersal sex biases among birds and mammals (following Greenwood 1980) and found some support in mammals but no support in birds (albeit with limited power; Mabry *et al.* 2013). These findings and ours support the view that the drivers of the directions of sex biases in dispersal are more complex than mating systems alone (Waser & Jones 1983; Clarke *et al.* 1997; Yáber & Rabenold 2002; Lawson Handley & Perrin 2007; Clutton-Brock & Lukas 2012).

Yáber & Rabenold (2002) extended Greenwood's (1980) mating system hypothesis by highlighting that sex differences in the incidence of natal dispersal in social species may principally reflect sex differences in the relative availability of breeding opportunities within and outside the natal group (the ‘breeding diversity’ hypothesis; see also Langen 1996; Richardson *et al.* 2002). The breeding diversity hypothesis offers a plausible explanation for several of the known examples of male-biased dispersal in cooperatively breeding birds: the unusual ability of females to breed as subordinates within their natal territory could account for clear female philopatry in the brown jay (Williams & Rabenold 2005) and white-throated magpie jay (Langen 1996; Berg *et al.* 2009), males dispersing earlier in life than females in the Australian magpie (Veltman & Carrick 1990; Hughes *et al.* 2003) and initial observations of females being more likely to delay dispersal than males in the Seychelles warbler (Richardson *et al.* 2002; but see Eikenaar *et al.* 2010 for an alternative explanation and a lack of sex-biased dispersal over the long term). That said, as male-biased dispersal is likely to facilitate female reproduction within the natal territory (by offering ready access to unrelated mates), it remains unclear whether these patterns reflect the envisaged effect of reproductive opportunities on dispersal patterns or the reverse (see Berg *et al.* 2009 for similar arguments).

As the breeding diversity hypothesis offers predictions regarding sex differences in the *incidence* of natal philopatry (or dispersal), caution is needed when applying it to white-browed sparrow weavers, in which the sex difference in dispersal documented here lies not in the *incidence* of natal philopatry, but in natal dispersal *distance*. That said, as genetic evidence confirms that female white-browed sparrow weavers never breed as subordinates (whether in their natal groups or not; Harrison *et al.* 2013a), differential reproductive benefits to subordinate females of staying on or near their natal territory cannot readily account for the evolution of male-biased dispersal in this species. Similarly, as the modest levels of extra-group paternity in white-browed sparrow weaver societies (12–18% of young; Harrison *et al.* 2013a,b) are principally sired by dominant males, male-biased dispersal in this species cannot be readily attributed to the availability of significant reproductive opportunities for floating males either, as has been hypothesized for other species (see Langen 1996; Williams & Rabenold 2005; Berg *et al.* 2009). Like the breeding diversity hypothesis, the inbreeding avoidance hypothesis (specifically, that female dispersal in social species may be favoured where male reproductive tenures are longer on average than the time that their daughters take to mature; cf Clutton-Brock 1989; see also Greenwood 1980; Liberg & von Schantz 1985) also seeks to explain the direction of sex bias in the *incidence* of natal philopatry (or dispersal) rather than in the *distance* that dispersing individuals travel. As such, while this hypothesis might help to explain sex biases in the incidence of philopatry in other cooperatively breeding birds (e.g. Berg *et al.* 2009; see also Clutton-Brock & Lukas 2012), it does not offer clear predictions relevant to explaining the sex difference in dispersal *distance* documented here. More broadly, while the potential benefits of avoiding inbreeding could certainly have favoured the evolution of a sex difference in dispersal distance in this species and others (Pusey 1987; Perrin & Mazalov 2000), it is not currently clear how such benefits could account specifically for the evolution of sex-reversed patterns of dispersal distance in white-browed sparrow weavers.

Sex differences in natal dispersal distance might be expected to arise as a consequence of sex differences in either the incidence or detectability of potential dispersal opportunities (in this case, dominance vacancies and contestable dominance positions) in the surrounding habitat. For example, subordinates of the sex that experiences a higher rate of dominance turnover may be more likely to encounter a dominance vacancy close to their natal group within a given time frame than the sex for which dominance turnovers are rare. This alone cannot readily explain the male-biased dispersal distances of white-browed sparrow weavers, however, as our long-term demographic data suggest that dominance turnover rates are similar for the sexes and, if anything, may be higher among males. More plausible, however, is the possibility that the observed sex difference in dispersal distance arises instead because other aspects of the species’ biology generate a sex difference in the distances over which the birds can detect dispersal opportunities (which we shall term the ‘opportunity detection’ hypothesis).

Sex differences in the distances over which dispersal opportunities can be detected could arise for at least two reasons, both of which might plausibly account for the male-biased dispersal distances documented here. First, if subordinates of one sex benefited from conducting extra-territorial prospecting forays over greater distances from their natal group (e.g. because distant forays offered males access to extra-group paternity, in addition to dispersal opportunities; Young *et al.* 2007), their longer-distance or more frequent forays might also leave them better placed to detect, and so contest, more distant natal dispersal opportunities. This could certainly be the case in white-browed sparrow weavers, as (i) subordinate males still resident in their natal groups are known to both conduct extra-territorial forays (Lewis 1982a) and secure extra-group paternity (Harrison *et al.* 2013b; albeit infrequently), and (ii) extra-group matings are known to occur over greater distances than both male and female dispersal (this study). As such, prospecting for distant extra-group matings might widen the spatial scale over which subordinate males are able to detect dispersal opportunities from their natal group. The same is unlikely to be true for subordinate females, as females never breed while subordinate (Harrison *et al.* 2013a), leaving them little cause to prospect specifically for extra-group matings. This argument is distinct from the role of extra-group paternity envisaged in the breeding diversity hypothesis, in which the potential for floating males to secure extra-group paternity is predicted to increase the *incidence* of male dispersal (see Yáber & Rabenold 2002; Williams & Rabenold 2005). Sex differences in the net benefits of distant prospecting could be of wider relevance to understanding sex biases in natal dispersal distances in the other species in which both sexes routinely delay dispersal. A second mechanism could also leave subordinate male white-browed sparrow weavers able to detect more distant dispersal opportunities than subordinate females. Dominant males sing a conspicuous dawn song each morning throughout the breeding season (Voigt *et al.* 2007; York *et al.* 2014), the absence of which (following the death or displacement of a resident dominant male) could reveal dominance vacancies or instability to an audience of subordinate males residing at considerable distances. In contrast, the lack of a comparable repertoire among dominant females (Voigt *et al.* 2007) may leave female vacancies rarely detectable beyond neighbouring groups.

## Conclusion

We have employed both observational and genetic data to demonstrate male-biased dispersal in a cooperatively breeding bird. This finding is important as it represents a rare reversal of the typical avian pattern of dispersal, taxonomically distinct from the handful of cooperatively breeding species in which male-biased dispersal has been documented to date (see Introduction). As such, our findings offer a new model system in which to evaluate the leading hypotheses for the evolution of dispersal sex biases in social species. That these hypotheses cannot readily account for the evolution of male-biased dispersal in white-browed sparrow weavers highlights the need for continued attention to alternative explanations for this enigmatic phenomenon. That our focal species exhibits no clear sex difference in the incidence of natal philopatry, coupled with sex-reversed patterns of dispersal distance, further highlights the need to both develop and test distinct hypotheses for the evolution of sex differences in the incidence of dispersal (or philopatry) and the distances over which it occurs (echoing Lawson Handley & Perrin 2007; Clutton-Brock & Lukas 2012; Dobson 2013). We suggest that attention to potential sex differences in the distances over which dispersal opportunities can be detected might usefully contribute to our understanding of the latter.

Recent studies have suggested that the most precise insights into patterns of dispersal are derived using both direct observational and indirect genetic data (Harris *et al.* 2009; Rollins *et al.* 2012; Griesser *et al.* 2013). In our study, both genetic and observational analyses revealed signals of male-biased dispersal, highlighting the potential for each approach to accurately identify unusual dispersal systems where necessity dictates their application in isolation. However, in the absence of corroboratory evidence from observational data, our findings also highlight the utility of drawing on multiple lines of genetic evidence when using population genetic structure analyses to draw inferences about sex differences in dispersal (Goudet *et al.* 2002). Genetic methods are likely to vary in their sensitivity (Banks & Peakall 2012), and the most robust inferences are likely to drawn when multiple lines of genetic evidence converge on the same conclusions with respect to dispersal patterns.

## Notes

J.E.Y. and A.J.Y. collected the data. X.A.H. and J.E.Y. analysed the data. X.A.H. and A.J.Y. wrote the manuscript. All authors commented on a final draft of the manuscript.

## Data accessibility

Data sets and R scripts used in this study are available from the Dryad data repository, DOI 10.5061/dryad.30qt0.

## Supporting Information

Fig S1 Decline in detection probability with increases in natal dispersal distance

Table S1 Four competing models describing the change in detectability of dispersal as dispersal distance increases.
