# Supplementary material for: Population genetic structure and direct observations reveal sex-reversed patterns of dispersal in a cooperative bird
Source: Mol Ecol. 2014 Nov 15;23(23):5740–55. doi: 10.1111/mec.12978 (PMC4265262; doi:10.1111/mec.12978)
Supplement: Fig S1 — Decline in detection probability with increases in natal dispersal distance [file mec0023-5740-SD1.docx]

Figure S1. Decline in detection probability with increases in natal dispersal distance. Blue line represents fitted value from a binomial glm where detection probability was a function of a 3^rd^ order polynomial for distance (see Table S1).
