# Supplementary material for: Population genetic structure and direct observations reveal sex-reversed patterns of dispersal in a cooperative bird
Source: Mol Ecol. 2014 Nov 15;23(23):5740–55. doi: 10.1111/mec.12978 (PMC4265262; doi:10.1111/mec.12978)
Supplement: Table S1 — Four competing models describing the change in detectability of dispersal as dispersal distance increases. [file mec0023-5740-SD2.docx]

| **Model** | **df** | **AICc** | **weight** |
| --- | --- | --- | --- |
| Distance^3^ | 4 | 908.323 | 1 |
| Distance^2^ | 3 | 4819.11 | 0 |
| Linear Distance | 2 | 7908.022 | 0 |
| Intercept Only | 1 | 308106.279 | 0 |

Table S1. Four competing models describing the change in detectability of dispersal as dispersal distance increases. Models are ranked by AICc. The best supported model contained a 3^rd^ order polynomial term for distance (see Figure S1)
